# Application Effect of Intelligent Monitoring of Emergency Nursing Process Reengineering in the Thrombolytic Therapy of Acute Myocardial Infarction

**DOI:** 10.1155/2021/3043289

**Published:** 2021-11-22

**Authors:** Xueqing Liu, Sufang Huang, Jing Cheng, Ying Zhang

**Affiliations:** ^1^Emergency Department, Tongji Hospital, Tongji Medical College, Huazhong University of Science and Technology, Wuhan, Hubei 430030, China; ^2^Cardiovascular Medicine, Tongji Hospital, Tongji Medical College, Huazhong University of Science and Technology, Wuhan, Hubei 430030, China

## Abstract

The application of emergency nursing process in intravenous thrombolytic therapy for patients with acute myocardial infarction was discussed. 100 patients with ST segment elevation myocardial infarction who met the inclusion and exclusion criteria were selected for intravenous thrombolysis. 50 patients with ST segment elevation myocardial infarction were treated from December 2018 to June 2019. The first-aid time and treatment effect of the two groups were compared. The results showed that the first aid time in the optimized process group was less than that in the conventional flow group (*P* < 0.05); the ECG rate was higher within 10 min than that in the conventional flow group (*P* < 0.05). It indicates that standardized and meticulous nursing procedures can avoid repetition and omissions and improve work efficiency. The application of the emergency care process in the emergency care of patients with acute myocardial infarction can win more rescue time and then improve the success rate of their rescue.

## 1. Introduction

In recent years, the incidence of acute myocardial infarction (AMI) in China has increased significantly. The occurrence of this disease is due to the atherosclerotic rupture in the patient's coronary artery and the platelet rapidly forms a thrombosis, thrombus to form a coronary artery in the cracking plaque surface, so that its myocardium is ischemic hypoxia. Acute myocardial infarction patients have not accepted timely, effective treatment, and their mortality is extremely high [1]. The most important treatment measures for acute myocardial infarction are reperfusion treatment, including direct perfusion arterial treatment and thrombolytic treatment. For hospitals without heart interpretation, it will select intravenous thrombolysis as a primary treatment, and the drug is administered intravenously to dissolve the drug in the patient's blood to improve the patient's coronary artery thrombosis [2, 3]. The method can be taken to alleviate the occurrence of myocardial infarction in patients and the myocardial function can also improve the role [4]. Therefore, the method has become an example of the treatment of AMI in the treatment of AMI in the treatment of emergency peripheral stent stenting (PCI) [5].

In clinical emergency, acute myocardial infarction is a relatively common condition because of the lack of coronary blood oxygen which caused rapid inaction dangerous condition and is very sharp after the sternum is very sharp and the mortality is extremely high. It is easy to lead to complications such as shock and heart failure, which seriously affect the physical and mental health of patients. The blood vessels that infarct the infarction are the key to the clinical success to treat patients with acute myocardial infarction. PCI (transdermal coronary intervention) is an effective method of clinical treatment of acute myocardial infarction patients and the “Guide of China throttle coronary intervention treatment” recommended that patients with acute myocardial infarction entering hospital emergency visit have been spent on the balloon to expand this process. Time should not exceed 90 min. This requires the clinical emergency nursing process to be efficient, high quality, and fast, so as to shorten the waiting time, preparation time, and transfer time of patients. Optimizing the emergency care process and helping the quality of emergency medical services have been further improved. Swain et al. analyzed the STEMI-data to the unloading (STEMI-DTU) test and then tested LV unloading on ischemia-reperfusion injury heart metabolism, heart metabolism, and mitochondrial function of acute myocardial infarction. The results showed that the duration before LV unloading and reperfusion was negatively correlated with the infarct size of patients with large ST segment elevation myocardial infarction. In the preclinical model, LV unloading reduces expression of ischemic hypoxia-sensitive protein and myocardial injury alone. Using inflammatory and blind teaser, TV-P improves the use of myocardial energy substrates and the mitochondrial structure including reperfusion IR or ECMO compared with the cardiolipin content. The mitochondrial function test from the infarction zone indicates that the intact mitochondrial structure includes a cardiolipin content, retains the electron transfer chain to the activity of the mitochondrial complex I, and reduces oxidative stress and TV-P-support reperfusion but not with IR or ECMO [6].

In summary, this study analyzed the clinical data of 100 acute myocardial infarction patients who were hospitalized in our hospital. It is proposed to explore the effect of optimization of emergency process on intravenous thrombolytic waiting time for acute myocardial infarction and optimize process group emergency time smaller than a conventional process group (*P* < 0.05); optimizing patient treatment processes set higher than the conventional process electrocardiogram 10 min group (*P* < 0.05). Indications to standardized detailed care procedures can avoid repeating and omissions and improve work efficiency. The rate of venous thrombolysis in the optimized process group was higher than that in the conventional process group, and the difference was statistically significant (*P* < 0.05). The results of this study show that optimizing the success rate of rescue rates in process groups, the incidence of complications and mortality is superior to the conventional process group and the difference is statistically significant (*P* < 0.05).

## 2. Research Methods

### 2.1. General Information

An individual hospital was treated with intravenous thrombolysis, consistent with STEMI patients incorporating the criteria. December 2018–June 2019 adopts venous thrombolytic treatment STEMI patients. 50 cases of STEMI patients using venous thrombolytic treatment in December 2020 were 50 cases of STEMI patients using venous thrombolytic therapy in December 2020 to optimize process groups. Incorporate standards: (1) continuous drama of chest pain >30 min, sustainable nitrate (NTG) does not alleviate. (2) Two or more of the two or more leading core map ST sections are lift ≥0.1 mv. (3) Abnormal increase in myocardial injury markers (in accordance with the first two conditions, it is determined that the diagnosis is STEMI and cannot delay the start of reperfusion treatment because of the results of the test of myocardial marker), such as creatine kinase (CK), CK isoenzyme (MB), cardiac troponin T (cTnT), cardiac troponin I (cTnI), and myoglobin Exclusion criteria: there is a thrombolytic contraindication. For the general data comparison of two groups, see Table 1.

### 2.2. Methods

#### 2.2.1. Conventional Process Group

The traditional emergency process is used. First, the patient has been admitted to the hospital, and the nursing staff follow the doctor's advice to conduct an electrocardiogram and the physician analyzes the report to conduct a preliminary diagnosis, inform the doctor of emergency consultation for confirmation, and then contact the coronary heart disease intensive care unit (CCU) to prepare for rescue; nursing staff follow the doctor's advice to carry out the rescue and treatment; after the preparation, the patient will be sent to the cardiology CCU. The CCU receives the patient after collecting 18-lead electrocardiogram blood and thrombolysis and communicates with the family to sign the consent and finally performs intravenous thrombolytic treatment.

#### 2.2.2. Optimizing Emergency Process Group

For all, the chest tightness chest pain patients were sent directly to the rescue room and inform the emergency medical physician to evaluate the rescue bed. For all patients with chest tightness and chest pain and patients with abdominal pain and shoulder pain who cannot be excluded as patients with suspected acute myocardial infarction, we should first pay attention to the inquiry, observation, and diagnosis of patients and carry out early routine ECG detection to make a correct risk assessment for patients. (1) Patients with suspected myocardial infarction need not be notified by the doctor for ECG detection, and 18-lead routine ECG collection shall be carried out immediately for preliminary judgment (2) Establish an ECG workstation or QQ group and WeChat group. First aid, intracardiac and CCU medical personnel, and ECG technicians communicate within the group. After the preliminary analysis of ECG, if the possibility of myocardial infarction cannot be ruled out, immediately contact the cardiologist for consultation. Upload the ECG to the chat group. ECG technicians and emergency consultation doctors can preliminarily analyze the ECG on the way and give rescue guidance. (3) With the oil-based pen or marker, the first electrocardiogram is used, which is convenient for later rescue; each ECG collects the chest and guarantees the true and accurate collection of data, and the data are comparable, lowering the electrocardiogram. The person is an error. The acute consultation physician confirmed myocardial infarction to achieve “three immediate actions”: (1) collect a full set of blood specimens, including blood routine, blood type, myocardial enzyme, myocardial marker, liver and kidney function, electrolyte, and coagulation function. (2) Immediate administration, mainly oral antithrombotic drugs, such as chewing, taking 300 mg aspirin, oral Plavix 300 mg dual anticoagulant, oral atorvastatin calcium 40 mg to strengthen lipid regulation; intravenous infusion of pantoprazole and other acid-inhibiting and stomach-protecting drugs; intravenous injection of heparin 3000∼5000 u. (3) Immediately communicate with their families, explain the condition and treatment plan, sign the venous thrombolytic consent, while notifying CCU to prepare for rescue, and notify the workers to do a good job.

The rescue personnel shall establish the first-aid mode of “fixed personnel, fixed post, fixed time, and fixed position”; (1) fixed personnel and fixed post, mainly composed of 1 doctor, 3 nurses, 1 nurse, and elevator staff. Doctors are responsible for physical examination, collecting patients' medical history, formulating rescue plans, and organizing rescue work; 1 nurse is responsible for the drug, resettlement of electrocardiography, lifeline monitoring, condition observation and judgment, etc. 2 nurses established a venous channel for patients' blood administration and notify the various departments and related personnel, etc. The caregiver is responsible for preparing water for patients to take medicine, assists patients to take medicine, timely checks the blood collection of nursing staff, and reasonably fixes the first aid equipment, also should reasonably and properly fix the first-aid equipment on the bed, and its central electric monitor shall be fixed at the head of the bed for easy observation; fix the defibrillator on the side of the bed or on the bed; the micropump is fixed at the end of the bed, and the oxygen cylinder is fixed at the head of the bed. At the same time, prepare for the transfer, and assist the nurse to contact the corresponding departments and personnel. The elevator worker immediately starts the green channel-specific elevator and stops to the 1st floor to prepare for quick transfer. (2) Timing: quantifying the rescue time and quantifying each step of the patient rescue; each operation is quantified with the project, strictly pressed within the time limit. (3) Positioning: set up a first aid kit, 6S management, according to the characteristics of the disease and rescue; place all items needed for rescuing patients in fixed positions according to type and quantity to form a “walking rescue vehicle.” With the patient's transport to any area, the instrument and equipment used in the rescue are placed in a first aid bed, and each item instrument is placed independently, preventing repeated time waste of the items, instruments, etc., saving valuable time.

### 2.3. Observations

#### 2.3.1. First Aid Time

The first aid time of acute myocardial infarction patients in two groups was observed. It mainly includes the time to ECG, the time to ECG report, the time to intravenous blood taking, the time to oral antithrombotic drug administration, the time to sign the consent form, and the time to intravenous thrombolysis.

#### 2.3.2. Therapeutic Effect

ECG rate within 10 min, intravenous thrombolysis rate within 30 min success rate of rescue, and incidence of complications and mortality were recorded in two groups.

### 2.4. Statistical Method

Statistical software SPSS19.0 was used to establish the database. The measurement data were described by ($\bar{x}\pm s$) and analyzed by T test. The counting data were expressed as percentage, and the difference was statistically significant if *P* < 0.05.

## 3. Result Analysis

### 3.1. Comparison of First Aid Time between the Two Groups

After receiving emergency care, there was no significant difference in the hospitalization time between the two groups (*P* > 0.05). The length of stay in the emergency room and the time from onset to receiving interventional therapy or thrombolytic therapy in the optimized group were shorter than those in the conventional group (*P* < 0.05), as shown in Table 2.

### 3.2. Comparison of Treatment Effect between the Two Groups

ECG rate within 10 min from admission to thrombolytic treatment intravenous thrombolytic rate within 30 min, success rate of rescue, and incidence of complications in the two groups: the time from admission to thrombolytic treatment in the optimized procedure group was significantly shorter than that in the control group, and the success rate of rescue in the optimized procedure group was higher than that in the conventional procedure group, with statistically significant differences between the two groups (*P* < 0.05). Comparing of bleeding incidence between the observation group and control group (*P* > 0.05), the difference was not statistically significant. See Table 3.

### 3.3. Satisfaction of Patients in the Two Groups

**Figure 1:**
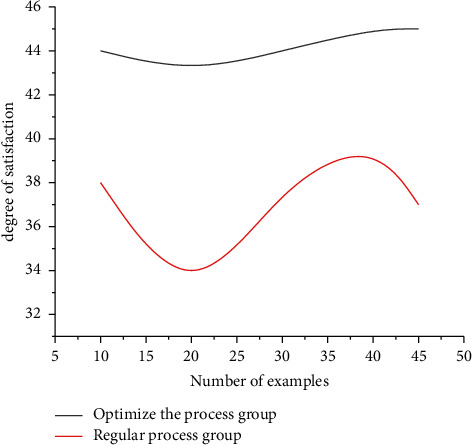
Comparison plot of satisfaction between the two groups.

Comparison of satisfaction between the two groups: satisfaction of the observation group was significantly higher than that of the control group and the difference was statistically significant (*P* < 0.05). See Figure 1.

## 4. Discussion

### 4.1. Effects of Optimizing Emergency Processes on Early ECG and Early Diagnosis Rate in Patients with Acute Myocardial Infarction

Sometimes, professional ECG technicians and cardiologists cannot accurately analyze the report at the first time. However, the emergency departments of primary medical institutions mostly have no professional cardiologists and the emergency medical staff have little contact with electrocardiogram and lack of knowledge. Therefore, they often cannot analyze or can only analyze typical common electrocardiogram or rely on the automatic report issued by the electrocardiogram machine to make a judgment. Therefore, ECG examination is often not considered in early patients, especially in patients with atypical myocardial infarction, resulting in early missed diagnosis and in-hospital delay of patients' reperfusion treatment. The significance of establishing and standardizing the application of the ECG workstation/network group is that even if emergency medical staff cannot analyze ECG, they can obtain professional ECG report results at the first time by collecting ECG and sending it to the workstation or network group, which greatly improves the enthusiasm of emergency doctors for early ECG examination. The early acquisition rate of ECG is improved [7]. For patients with suspected myocardial infarction, emergency nurses immediately and routinely collect 18-lead electrocardiogram without doctors' orders which not only improves the early ECG collection rate but also greatly reduces the rate of missed diagnosis and further shortens the waiting time for reperfusion treatment [8]. The results of this study showed that the first aid time of the optimized procedure group was shorter than that of the conventional procedure group (*P* < 0.05). The electrocardiogram rate within 10 min in the optimized procedure group was higher than that in the conventional procedure group (*P* < 0.05). It shows that standardized and meticulous nursing procedures can avoid repetition and omission and improve work efficiency.

### 4.2. Effect of Optimizing Emergency Procedures on Intravenous Thrombolytic Rate of Patients with Acute Myocardial Infarction within 30 Minutes of Visit

This study showed that the rate of intravenous thrombolysis within 30 min in the optimized procedure group was higher than that in the conventional procedure group, and the difference was statistically significant (*P* < 0.05). At present, the study of STEMI focuses more on the emergency PCI, and only a handful of similar emergency mode was used in the study of emergency PCI. For intravenous thrombolytic therapy, the first-aid mode of “fixed personnel, fixed post, fixed time, and fixed position” is a cooperative mode of medical integration. Personnel at all levels cooperate closely, and the process is seamlessly connected, which can give full play to the role of everyone at each post and give full play to the best role of each measure to the greatest extent. Quantification and strict execution of the first aid time for every step, operation, and project necessary for rescue, as well as the establishment of the first aid kit and 6S management of items make all needed items close at hand and clear at a glance, which reduces the time wasted by running around and searching and gives play to the remarkable effect of this first aid mode [9]. The advance of blood taking, drug administration, and thrombolytic consent signing, as well as the sufficient manpower in the emergency department, can effectively avoid the lack of nursing staff in the ward after admission, the need to execute a large number of doctor's orders and rescue measures in a short time, and the hospital delay caused by the delayed implementation, which can effectively shorten the first aid time of patients and improve the treatment effect.

### 4.3. Influence of Optimizing Emergency Procedures on the Success Rate of Rescue, Complications, and Mortality of Patients with Acute Myocardial Infarction

The results of this study showed that the success rate of rescue, incidence of complications, and mortality of patients in the optimized procedure group were better than those in the conventional procedure group, with statistical significance (*P* < 0.05). Under the joint action of various emergency procedures optimization measures, the rate of early diagnosis of patients has been greatly improved, avoiding misdiagnosis and misdiagnosis of inappropriate specialty, and even the further deterioration and influence of the disease caused by the wrong treatment plan. Large hospitals are connected with secondary hospitals, communities, township hospitals, and other specialized doctors to form remote ECG reporting center and consultation center to help diagnose and guide rescue at the first time [10]. After the onset of the disease, patients can receive timely accurate and effective treatment in the nearest community or health center at home. They can be transferred to large hospitals for further treatment at the same time of reperfusion rescue or after the condition is controlled, which can shorten the time delay on the way to large hospitals and greatly advance the time of reperfusion treatment. Early reperfusion saved the dying myocardium, reduced the infarction, and prevented left ventricular remodeling. The optimization of the emergency nursing process can improve the success rate of treatment, reduce recurrence, high cure rate, light complications, and low mortality, and improve the prognosis and quality of life of patients.

## 5. Conclusions

The results of this study confirmed that the implementation of emergency nursing process can enable doctors to treat myocardial infarction within the “time window” of treatment and shorten the time of acute myocardial infarction in patients with acute myocardial infarction. It can also reduce the recurrence rate and mortality of acute myocardial infarction, reduce complications, and protect cardiac function. In addition, it can also reduce the hospitalization time of patients, reduce medical expenses, and improve patients' satisfaction with nursing. These are of great significance to improve nursing quality and work efficiency. It is a management model worthy of clinical promotion in grass-roots hospitals.

## Figures and Tables

**Table 1:** Comparison of general data in the two groups ($\bar{x}\pm s$).

Group	Example number	Sex		Age (year)	Site of myocardial infarction					
		Man	Woman		Antetheca	Paries inferior	Lower wall + right chamber	Lower wall + rear wall	Lower wall + front wall	Front wall + high side wall
Regular process group	50	32	18	62.8 ± 11.4	36	4	2	5	2	1

Optimize the process group	50	34	16	66.1 ± 14.2	34	3	3	5	3	2

Note: the anterior wall of the myocardial infarction site includes the anterior interwall, restricted anterior wall, extensive anterior wall, etc. *P* > 0.05.

**Table 2:** Comparison of first aid time between the two groups $\left(\bar{x}\pm s\right)$.

Group	Example number	See an electrocardiogram Time	See an electrocardiogram report time	Blood collection time to the vein	Visit to oral thrombolytic drug time	Visit the doctor until signing the consent form	Visit the vein bolt time
Regular process group	50	7.7 ± 4.9	8.1 ± 5.2	9.1 ± 5.2	22.5 ± 6.5	26.0 ± 6.4	28.2 ± 6.2
Optimize the process group	50	4.4 ± 2.8	4.5 ± 3.0	4.7 ± 2.9	5.8 ± 2.7	17.7 ± 6.3	19.5 ± 6.1
*t*		5.01	5.10	6.47	18.12	7.73	7.87
*P*		<0.05	<0.05	<0.05	<0.05	<0.05	<0.05

**Table 3:** Comparison of the treatment effect between the two patient groups.

Group	Example number	The ECG rate within 10 min	Rate of venous thrombolysis within 30 min	Rescue success rate	Rescue success rate	Death rate
Regular process group	50	31 (62)	30 (60)	36 (2)	20 (40)	4 (8)
Optimize the process group	50	47 (94)	46 (92)	48 (96)	5 (10)	0 (0)
*x* ^2^						
*P*		<0.05	<0.05	<0.05	<0.05	<0.05

## Data Availability

The data used to support the findings of this study are available from the corresponding author upon request.
